# Summer Time

**Published:** 1919-06-07

**Authors:** 


### The Hospital

The Workers' Newspaper of Administrative Medicine and Institutional Life, Administration, National Insurance and Health.

No. 1722, Vol. LXVI. SATURDAY, JUNE 7, 1919. PRICE TWOPENCE

### SUMMER TIME.

Man is a diurnal animal. The sunken position of his eyes and the relatively small size of his irides and pupil render it certain that he and his immediate ancestry are adapted to a life of activity during daylight and of inactivity in the dark, and to such a life he must have been restricted until he invented artificial means of lighting. In winter in these latitudes the daylight hours are short, and it is probable that man's habitations did not spread as far north as this until he had invented artificial light. Primitive artificial lights were inefficient, as those who remember the dip candle and the rush-light will acknowledge, and therefore man made the most of the daylight hours. For more than six months in the year men rose at least as early as the sun, and arranged their hours of work and meals accordingly. The curious student of manners notes a gradual advance in the hour of taking the chief meal of the day. What was dinner-time to our Saxon ancestors is known only to the learned, but in Queen Elizabeth's days it was eleven in the forenoon. A century later, in the time of Charles II., it had advanced an hour. In the eighteenth century the advance was more rapid. In his early days Dr. Johnson dined how and when he would; but at the height of his fame, when he dined with "the great," his dinner-hour was between five and six. By Napoleon's time the hour of dinner had advanced to six, and in the days of Thackeray and Dickens half-past six was ultra-fashionable. In the middle of the nineteenth century dinner-parties were summoned for seven, and the time gradually crept on until Edward VII. dined at a quarter to nine.

Such an advance in the hour of dinner led naturally to an advance in the hour of retiring to rest. Fashionable people went later and later to bed, rose later and later in the morning, and lived more and more by artificial light. Living less in sunlight, their complexions suffered. To improve their complexions, fashionable ladies resorted more and more to cosmetics. To disguise the use of cosmetics, they resorted more and more to artificial light. Would-be fashionable people imitated those above them in rank, and thus for one reason or another the hour of beginning activity approached nearer and nearer to noon, and the hour of retiring was delayed until past midnight, and thus man, from being a purely diurnal animal, became crepuscular, and more and more nocturnal.

At length the world awoke to the unnaturalness and inconvenience of this habit, and desired to alter it; but, instead of taking the bull by the horns and putting back the hour of dinner, which would have been naturally followed by a revision of the hours of our other activities, we resorted to a trick, and tried to deceive ourselves and each other by putting on the clock, a childish device, but one that has been so far fairly successful. We now put the clock on one hour, but if we allow ourselves to slide into the old habit of advancing the dinner hour we shall be compelled in a few years, if we wish to produce the same effect, to inaugurate summer time by putting the clock two hours forward; and who knows what further absurdities may not be in store for us?

Man is eminently adaptable, and he can modify his own habits pretty much as he chooses; but he cannot alter the whole order of nature to suit his notions, and farmers and gardeners cannot reconcile themselves nor the objects of their care to an artificial summer time. Cows are accustomed to be milked, and all live-stock to be fed, at certain hours by the sun, not by the clock; and when these hours are arbitrarily altered, the stock do not thrive as well, nor do the cows give their milk as willingly. The gardener cannot postpone the watering of his plants until the sun has lost its fierceness, but must water them in the scorching heat. The inconsiderate tides persist in rising and falling according to the movements of the moon, and the fisherman and the pilot must regulate their actions by two clocks recording different time. And all these inconveniences must be suffered because men prefer to deceive themselves by a transparent untruth instead of going straightforward to work and rising an hour earlier. Truly we are queer creatures. But what is the root of it all? Whence comes this tendency to postpone all the functions of life to a later and later hour in the day? Why have we been for centuries gradually transforming ourselves from diurnal into nocturnal animals? To have endured so long and to have persisted so steadily, the cause must lie deep, and be common to the majority of mankind. What is it? Procrastination? Laziness? Indolence? Perhaps. But we are to remember that for many centuries the population of this country, in common with that of the rest of Europe, was stationary. When, in the sixteenth century, it began to increase, the struggle for life became more severe. To earn a livelihood a man must do more work; more work meant more hours of work, and these hours were naturally added at the end of the day, when it was found that the daily task must otherwise be left unfinished. Late hours of work meant later retiring to rest; and later retiring to rest meant later waking in the morning, and so the hours of waking life crept down the day. If our numbers were to be reduced to one-tenth of what they are, and our requirements were no more than they were four hundred years ago, we should still dine at noon, gnawing the beef bones in the absence of forks, and pitching them to the dogs nestling in the straw and rushes that did duty for carpets. We should not need to put our clocks an hour in advance of Greenwich mean time, a fortunate circumstance, since we should have no clocks to alter.

